# Cross-cultural adaptation, content validity, and item analysis of the Objective and Subjective Knowledge and HIV Testing Scale for the Brazilian population

**DOI:** 10.47626/2237-6089-2022-0519

**Published:** 2023-06-27

**Authors:** Rafaella Alves Silva, Jonathan Leonardo Gonçalves Prudencio, Edson Zangiacomi Martinez, Miriane Lucindo Zucoloto

**Affiliations:** 1 Escola de Enfermagem de Ribeirão Preto Universidade de São Paulo Ribeirão Preto SP Brazil Escola de Enfermagem de Ribeirão Preto, Universidade de São Paulo (USP), Ribeirão Preto, SP, Brazil.; 2 Faculdade de Medicina de Ribeirão Preto USP Ribeirão Preto SP Brazil Faculdade de Medicina de Ribeirão Preto, USP, Ribeirão Preto, SP, Brazil.

**Keywords:** Knowledge, attitudes, cross-cultural adaptation, human immunodeficiency viruses (HIV

## Abstract

**Objectives:**

To propose a Portuguese version of the Objective and Subjective Knowledge and HIV Testing Scale (OSK-HIV-TS), assess its content validity, and perform item analysis after administration to a sample of undergraduate students.

**Methods:**

Three translators translated the OSK-HIV-TS into Portuguese. Judges evaluated each item of a consensus version of the translated instrument for semantic, idiomatic, cultural, and conceptual equivalence. A consensus committee reviewed a back-translation against the original version of the OSK-HIV-TS. Content validity was calculated with the content validity index (CVI) and item analysis was conducted using Classical Test Theory (CTT).

**Results:**

The translated scale achieved semantic, idiomatic, conceptual, and cultural equivalence to the original version. A total of 491 undergraduate students participated and the distribution of students’ responses to the OSK-HIV-TS revealed a high proportion of correct answers. All items were classified as easy or very easy and only item 16 was classified having strong discrimination power according to the discrimination index.

**Conclusion:**

The OSK-HIV-TS is a novel instrument in the Brazilian literature for assessing human immunodeficiency virus (HIV) knowledge and should inspire more research into HIV/acquired immunodeficiency syndrome (AIDS) behavior and associated factors, which, despite being essential and necessary, is still lacking in the Brazilian literature.

## Introduction

Human immunodeficiency virus (HIV) infection has been considered a global epidemic since the end of the last century and a public health issue, especially in low and medium-income countries. The number of AIDS cases in Brazil has been stable over the past few years but, despite adoption of control strategies, the incidence of HIV-infected persons is increasing and is concentrated in certain population groups. The Brazilian Ministry of Health epidemiological bulletin on HIV/AIDS, covering cases notified until December 2020,^1^ describes an increase in the AIDS detection rate, mainly observed among young men. In 2019, the highest detection rate was 52 cases per 100 thousand inhabitants, which occurred among individuals aged 25 to 29 years. Increases of 64.9% and 74.8% were observed in the 15 to 19 and 20 to 24 age groups, respectively.

According to several studies,^2 - 5^ the disease’s multiplication in young people can occur due to biological factors, such as an exposed histological system due to development of the reproductive system, psychological factors, due to impaired personal relations, formation and questions regarding sexuality, which can lead to varying partners, and social aspects, such as lack of knowledge regarding HIV, social inequality, prejudice, socioeconomic status, and functional and structural conditions of the health system.

In view of this, Cabral et al.^6^ and Dzah et al.^7^ point out that lack of knowledge about HIV has been identified as a risk factor for dissemination of the virus among the young population and suggest that it is important to conduct more studies about the topic in different populations. Bednarsh and Eklund^8^ demonstrate that prevention is based mainly on promotion of behavioral changes and it is therefore necessary to conduct research regarding knowledge, attitudes, behaviors, practices, and perception. Thus, it is believed that sufficient knowledge may have implications for HIV prevention, such as decreased risk behaviors, better perception of exposure, more frequent testing, lower exposure, and adoption of safer behaviors. However, evaluation of psychometric constructs such as knowledge about HIV is limited in Brazil, in particular because of the scarcity of adapted and validated measurement scales for this purpose.

There are some scales in the literature for measurement of knowledge about HIV, such as the AIDS Attitude Scale,^9^ the scale of attitudes concerning AIDS,^10^ the Objective and Subjective Knowledge and HIV Testing Scale (OSK-HIV-TS), for use among college students,^11^ and the HIV/AIDS attitudes scale.^12^ However, none of these scales have a Portuguese version and there are no instruments available for assessing the HIV knowledge construct that can be used in studies in Brazil. Of the scales proposed to date, the OSK-HIV-TS, developed by Hou^11^ for use with college student populations has proven to be an interesting scale because it provides a more complete measure of knowledge (both objective and subjective). The original version of this scale is in English. The OSK-HIV-TS^11^ was originally developed for use with college students and comprises 16 items; 11 general questions about HIV/AIDS and five specific questions that assess general knowledge about the topic (subjective). Since it is a knowledge scale, the response categories are “true” and “false.”

Therefore, considering the lack of available scales on this issue in Brazil and the relevance to the field of cross-cultural adaptations of scales on HIV knowledge, the objective of this study is to present the steps of translation into Brazilian Portuguese and cross-cultural adaptation, assessment of content validity, and item analysis of the OSK-HIV-TS when administered to a sample of undergraduate students.

## Methods

### Translation, cross-cultural adaptation, and content validity

Cross-cultural adaptation of the scale to Brazilian Portuguese followed the methodology proposed by Ferrer et al.^13^ Initially, translations were conducted independently by three bilingual translators (whose native language was Portuguese and second language was English), obtaining three Brazilian Portuguese versions. The translators were experienced in translating psychometric scales and were instructed on the goals of the translation and informed about the target population of the study. Their versions were subsequently synthesized to construct a first version of the instrument in Brazilian Portuguese by the researchers responsible. Following Fehring’s criteria,^14^ five judges (health professionals with experience in validation studies) were selected to form a specialist committee to evaluate the items and suggest alterations to improve comprehension by the Brazilian population. In addition, these judges evaluated each item of the translated instrument individually for semantic, idiomatic, cultural, and conceptual equivalence. Semantic equivalence refers to the meaning of words in terms of vocabulary and grammar, idiomatic equivalence is related to the equivalence of expressions and “meanings” in different languages, cultural equivalence is related to adaptation to the context of the study’s target public, and conceptual equivalence is related to maintenance of the original instrument’s concepts. After this step, the researchers obtained a final version, considering the judges’ suggestions and the scale applicability criteria in the target population.

A panel of five specialists with experience in cross-cultural adaptation and validation of instruments independently assessed the content of the OSK-HIV-TS items to evaluate its objectivity and relevance. These specialists were informed about the study objectives and were asked to classify each item of the translated instrument as adequate or inadequate for use in the target population. Suggestions for modifications and simplification were requested when necessary. A content validity index (CVI) was calculated for each item, considering that CVI ≤ 0.70 (i.e., 30% or more of the judges classified the item as inadequate) is indicative that a new translation is needed.^15^

As a final step, a back-translation was carried out by a bilingual translator who had no previous knowledge of the original version of the scale. This step is important to confirm that all adapted items are equivalent to the instrument’s original proposal.

### Data collection in the target population

The Portuguese version of the OSK-HIV-TS was administered to a sample of undergraduate students in an open web survey. The study population comprised students enrolled on courses run by the eight units of the Universidade de São Paulo (USP), Ribeirão Preto Campus. An electronic version of the data collection instrument was developed on the Research Electronic Data Capture (REDCap) platform (http://project-redcap.org).^16^

An invitation to participate containing a link to the online survey was sent to all students by e-mail or messaging applications. The first questions on the survey were asked after agreement to participate had been given and were about the inclusion criteria (age ≥ 18 years and enrollment on an undergraduate course at one of the university’s eight units). Data collection was carried out from March to May 2021, and the average time taken to answer the survey was approximately 15 minutes.

### OSK-HIV-TS item analysis

Participants were classified based on their performance on the instrument using Kelley’s^17^ suggestion, which is to estimate cut-off points using the 27% of participants with the highest and lowest scores on the instrument. For the objective dimension of the OSK-HIV-TS, comprising 16 items, the cut-off points adopted to select the participants with the best and worst performances were 16 correct answers and ≤ 13 correct answers, respectively. Classical test theory (CTT) was used to assess the quality of OSK-HIV-TS items, using the facility and discrimination indices as parameters.^18^ For the facility index, the degree of facility of items is estimated through the proportion of correct answers, by which each item can be classified as “very easy” (proportion of correct answers from 80 to 100%), “easy” (60 to 80%), “medium difficulty” (40-60%), “difficult” (20-40%), or “very difficult” (0-20%). The discrimination index allows researchers to assess how well an item can distinguish between respondents who gave the best and worst responses on the instrument. In other words, the bigger the difference in the number of correct responses between participants with best and worse performance, the greater the item’s discrimination power. Discrimination index results from 0 to 30% represent weak discrimination, rates from 30 to 60% indicate moderate discrimination, and indices from 60 to 100% demonstrate strong discrimination.^19^ The goal of this analysis is therefore to find easy items with high discriminatory power (i.e., questions that are most likely to be answered correctly).

### Ethical considerations

The study was approved by the Research Ethics Committee at the Hospital das Clínicas, Faculdade de Medicina de Ribeirão Preto (CAAE: 31049220.4.0000.5440) and all ethical principles were respected. The informed consent form was presented on the first page of the online form and the survey only began if the participant agreed to participate in the study by clicking the option “I agree to the consent form.” The option “I prefer not to answer” was included for all questions on the online form and participants had the option to stop participating at any time.

## Results

After completing the procedures of translation and cross-cultural adaptation as described in the Methods section, with assessment of all types of equivalence in the translation process and achieving CVI values ≥ 0.75 for all items, the final Brazilian Portuguese version of OSK-HIV-TS is presented in Table 1 . The original English language version is also presented for comparison and some changes made to items to update concepts about HIV/AIDS are shown.

**Table 1:** Original version of the ^10^ OSK-HIV-TS and final version adapted to Brazilian Portuguese, Brazil, 2021

Item	Original version	Portuguese version	Updates
Objective knowledge		Conhecimento objetivo	
1	Teenagers and young adults are at high risk of being infected with HIV. (T)	Adolescentes e adultos jovens são considerados de alto risco para infecção pelo HIV. (V)	Not necessary.
2	HIV/AIDS can be transmitted through mosquito bites. (F)	O HIV pode ser transmitido pela picada de mosquitos. (F)	Not necessary.
3	HIV/AIDS can be transmitted if uninfected person donates his/her blood. (F)	Uma pessoa recém infectada pelo HIV pode transmiti-lo ao doar sangue. (V)	The judges suggested measuring the concept of HIV and blood donation considering aspects inherent to the immunological window.
4	A person can get HIV from oral sex. (T)	Uma pessoa pode contrair HIV por sexo oral. (V)	Not necessary.
5	Taking an HIV test 1 week after having sex can tell a person if he/she has HIV. (F)	Fazer um teste/exame de HIV nos primeiros dias após a relação sexual desprotegida já pode dizer a uma pessoa se ela tem HIV. (F)	Due to the evolution of HIV tests and the consequent reduction in the immunological window period, this question was reformulated.
6	A person can get HIV even if he/she has only one unprotected sexual encounter with an HIV-infected person. (T)	Uma pessoa pode contrair o HIV mesmo que tenha apenas uma única relação sexual desprotegida com uma pessoa infectada pelo HIV. (V)	Not necessary.
7	In general, it takes 3-6 months for a person with HIV to develop AIDS. (F)	Geralmente leva poucos dias para uma pessoa infectada pelo HIV vir a desenvolver a AIDS. (F)	Considering that it takes a few weeks for an individual to develop the disease after infection, this question remains false if we assume that this does not occur in the first few days.
8	HIV testing cannot be done unless you request or agree to have it done. (T)	O teste de HIV não pode ser feito sem a solicitação ou a concordância da pessoa a ser testada. (V)	Not necessary.
9	A person would know if he/she had been infected with HIV. (F)	Uma pessoa saberia se tivesse sido infectada pelo HIV. (F)	Not necessary.
10	A person with HIV can look and feel healthy. (T)	Uma pessoa com HIV pode parecer saudável e se sentir bem. (V)	Not necessary.
11	A pregnant woman with HIV can pass the virus to her baby (fetus). (T)	Uma mulher grávida portadora de HIV pode passar o vírus para o bebê (feto). (V)	Not necessary.
12	It is harder for women to get HIV from men than for men to get HIV from women. (F)	É mais difícil mulheres pegarem o HIV dos homens do que os homens pegarem HIV das mulheres. (F)	Not necessary.
13	HIV testing is usually anonymous and/or confidential. (T)	O teste de HIV geralmente é anônimo e/ou confidencia. (V)	Not necessary.
14	Douching after sex can keep a woman from getting HIV. (F)	Usar a ducha após o sexo pode impedir uma pessoa de contrair HIV. (F)	The judges suggested removing the gender of the question, considering other types of sexual intercourse than just vaginal.
15	Any time blood is drawn, it is tested for HIV. (F)	Em todos os exames de sangue que uma pessoa faz, é feito teste para o HIV. (F)	Not necessary.
16	It takes a couple of weeks or months from infection with HIV for detection by testing. (T)	A infecção pelo HIV pode levar algumas semanas ou meses para conseguir ser detectada pelos testes/exames. (V)	The expression “it takes” was changed to “can take” in Portuguese due to the evolution of tests for HIV detection.
Subjective knowledge		Conhecimento subjetivo	
1	In general, how do you rate your knowledge about HIV/AIDS? (Excellent/Good/Fair/Bad/I don’t know)	Em geral, como você classifica o seu conhecimento sobre o HIV/AIDS? (Excelente/Bom/Razoável/Ruim/Não sei)	Not necessary.
2	In general, how do you rate your knowledge specifically related to HIV testing? (Excellent/Good/Fair/Bad/I don’t know)	Em geral, como você classifica o seu conhecimento especificamente relacionado aos testes de HIV? (Excelente/Bom/Razoável/Ruim/Não sei)	Not necessary.

AIDS = acquired immunodeficiency syndrome; F = false; HIV = human immunodeficiency virus; OSK-HIV-TS = Objective and Subjective Knowledge and HIV Testing Scale; T/V = true.

A total of 491 undergraduate students enrolled at eight USP units at the Ribeirão Preto Campus participated in the study. Of these, 61.5% were female and 0.6% declared they had a non-binary identity, while 6.5% of the participants did not answer the questions about sex and gender. With regard to sexual orientation, 61.7% declared they were heterosexual, followed by 23.2% bisexual and 11.4% homosexual.

The distribution of students’ responses to the OSK-HIV-TS is presented in Table 2 .

**Table 2:** Proportion of undergraduate students’ (n = 525) responses to each item of the OSK-HIV-TS, Ribeirão Preto, São Paulo, 2021

Item	OSK-HIV-TS		Male n (%)	Female n (%)	Total n (%)
Objective knowledge					
1	Teenagers and young adults are at high risk of being infected with HIV.	**True**	172 (91.0)	294 (97.4)	466 (94.9)
		False	17 (9.0)	8 (2.6)	25 (5.1)
2	HIV/AIDS can be transmitted through mosquito bites.	**False**	184 (97.4)	297 (98.3)	481 (98.0)
		True	5 (2.6)	5 (1.7)	10 (2.0)
3	A newly infected person with HIV can spread the virus by donating blood.	**True**	148 (78.3)	251 (83.1)	399 (81.3)
		False	41 (21.7)	51 (16.9)	92 (18.7)
4	A person can get HIV from oral sex.	**True**	152 (80.4)	259 (85.8)	411 (83.7)
		False	37 (19.6)	43 (14.2)	80 (16.3)
5	Taking an HIV test within the first few days after unprotected sex can already tell a person if they have HIV.	**False**	155 (82.0)	222 (73.8)	377 (76.9)
		True	34 (18.0)	79 (26.2)	114 (23.1)
6	A person can get HIV even if he/she has only one unprotected sexual encounter with an HIV-infected person.	**True**	187 (98.9)	299 (99.0)	486 (99.0)
		False	2 (1.1)	3 (1.0)	5 (1.0)
7	In general, an HIV-positive person usually develops AIDS within a few days.	**False**	160 (84.7)	258 (85.4)	418 (85.1)
		True	29 (15.3)	44 (14.6)	73 (14.9)
8	HIV testing cannot be done unless you request or agree to have it done.	**True**	158 (83.0)	264 (87.4)	422 (85.9)
		False	31 (16.4)	38 (12.6)	69 (14.1)
9	A person would know if he/she had been infected with HIV.	**False**	185 (97.9)	300 (99.3)	485 (98.8)
		True	4 (2.1)	2 (0.7)	6 (1.2)
10	A person with HIV can look and feel healthy.	**True**	186 (98.4)	292 (97.0)	478 (97.6)
		False	3 (1.6)	9 (3.0)	12 (2.4)
11	A pregnant woman with HIV can pass the virus to her baby (fetus).	**True**	173 (91.5)	285 (94.4)	458 (93.3)
		False	16 (8.5)	17 (5.6)	33 (6.7)
12	It is harder for women to get HIV from men than for men to get HIV from women.	**False**	169 (89.4)	273 (90.4)	442 (90.0)
		True	20 (10.6)	29 (9.6)	49 (10.0)
13	HIV testing is usually anonymous and/or confidential.	**True**	175 (92.6)	271 (90.0)	446 (91.0)
		False	14 (7.4)	30 (10.0)	44 (9.0)
14	Douching after sex can keep a person from getting HIV.	**False**	185 (97.9)	297 (98.3)	482 (98.2)
		True	4 (2.1)	5 (1.7)	9 (1.8)
15	Any time blood is drawn, it is tested for HIV.	**False**	150 (79.8)	281 (93.0)	431 (88.0)
		True	38 (20.2)	21 (7.0)	59 (12.0)
16	It takes a couple of weeks or months from infection with HIV for detection by testing.	**True**	142 (75.5)	236 (78.4)	378 (77.3)
		False	46 (24.5)	65 (21.6)	111 (22.7)
**Subjective knowledge**					
1	In general, how do you rate your knowledge about HIV/AIDS?	Excellent Good Fair Bad I don’t know	15 (8.0) 76 (40.4) 87 (46.3) 10 (5.3) -	5 (1.7) 124 (41.1) 141 (46.7) 32 (10.6) -	20 (4.1) 200 (40.8) 228 (46.5) 42 (8.6) -
2	In general, how do you rate your knowledge specifically related to HIV testing?	Excellent Good Fair Bad I don’t know	10 (5.3) 41 (21.8) 77 (41.0) 56 (29.8) 4 (2.1)	2 (0.7) 41 (13.6) 135 (44.7) 119 (39.4) 5 (1.7)	12 (2.4) 82 (16.7) 212 (43.3) 175 (35.7) 9 (1.8)

AIDS = acquired immunodeficiency syndrome; HIV = human immunodeficiency virus; OSK-HIV-TS = Objective and Subjective Knowledge and HIV Testing Scale.

The correct answer to each item is in bold font.

The distribution of the students’ responses to the OSK-HIV-TS revealed a high proportion of correct answers. The item about contracting HIV through a single unprotected sexual contact with an HIV-positive person had the highest percentage of correct answers in the sample. On the other hand, the item with the lowest percentage of correct answers was about HIV testing in the first few days after unprotected sexual intercourse (window period). Both sexes had similar frequencies of correct answers for most items. However, it was notable that males had a higher frequency of correct answers for item 15 (about HIV testing any time blood is drawn) and females had higher frequencies of correct answers for item 5 (about the window period). It was also noteworthy that in the individual assessments on subjective knowledge, most students classified their knowledge about HIV in general and about tests as reasonable.

The quality of items of the OSK-HIV-TS for the Brazilian population, according to facility and discrimination indices obtained with CTT, is presented in Table 3 .

**Table 3:** Quality of items comprising the objective dimension of the Objective and Subjective Knowledge and HIV Testing Scale (OSK-HIV-TS) according to facility and discrimination indices obtained using Classical Test Theory (CTT) with a sample of undergraduate students (n = 491), Ribeirão Preto, São Paulo, 2021

Item	Worst (%)	Best (%)	Discrimination index (%)	Facility index (%)
1	88.3	100.0	11.7	94.9
2	93.7	100.0	6.3	98.0
3	72.1	100.0	27.9	81.3
4	70.3	100.0	29.7	83.7
5	41.4	100.0	58.6	76.8
6	96.4	100.0	3.6	99.0
7	56.8	100.0	43.2	85.1
8	73.9	100.0	26.1	85.9
9	94.6	100.0	5.4	98.8
10	90.1	100.0	9.9	97.5
11	90.1	100.0	9.9	93.3
12	77.5	100.0	22.5	90.0
13	83.8	100.0	16.2	90.8
14	94.6	100.0	5.4	98.2
15	69.4	100.0	30.6	87.8
16	38.7	100.0	61.3*	77.4

* Item with the highest discrimination index.

All items were classified as easy or very easy when applied to the sample of undergraduate students. Only item 16 was classified as having strong discrimination.

## Discussion

Cross-cultural adaptation of an instrument is performed when there is a need to develop specific measures for countries other than where the scale was originally developed. This process needs rigorous methodology supported by a theoretical framework that can guide the translation, adaptation, and validation steps.^20^ Thus, having conducted all of the steps presented above, the resulting Portuguese version of the OSK-HIV-TS can be considered adequate for administration to Brazilian populations and to support new studies in which assessment of knowledge about HIV/AIDS would be useful.

According to the high frequency of correct answers, we can classify the students’ general knowledge about HIV as high. However, it is of concern that around 20% of these students did not know about the window period. Few studies have assessed knowledge about HIV in the young Brazilian population and those that do exist do not cover the subject of the window period. In addition, studies that further assess knowledge about the HIV window period in Brazil are focused on the population of men who have sex with men or blood donors. Knowing about the window period is important to interrupt transmission of the disease and is a relevant topic to include in instruments and models for assessing knowledge about STIs. As such, the OSK-HIV-TS contributes to the literature by including a question specifically about the window period in its items on general knowledge about HIV.

In our sample of undergraduate students, the item quality analysis only classified item 16 as having strong discrimination power and all items were classified as easy or very easy according to the facility index. More studies are needed to evaluate item content in order to confirm the items’ low discrimination power and suggest changes to improve the instrument.

Although the original proposal focused on college students, we believe that the version proposed here may be suitable for administration to Brazilian populations in different contexts, due to its simple and easy-to-understand language. However, we suggest that pre-tests of comprehensibility and applicability in the target population should always carried out before each application, so that the quality of the data obtained is guaranteed, especially in studies that involve data collection using the self-administered format. In addition, we hope that this scale can motivate further studies on behavior towards HIV/AIDS and associated factors, which are still scarce in the Brazilian literature, despite being urgent and necessary.

## Conclusion

The OSK-HIV-TS is a novel instrument for assessing HIV knowledge in the Brazilian literature that should inspire more research on HIV/AIDS behavior and associated factors, which, despite being essential and necessary, are still lacking in the Brazilian literature. According to all of the steps presented, the OSK-HIV-TS can be considered adequate for administration to Brazilian undergraduate students. Further studies examining other psychometric properties of the scale would be a welcome addition to overall assessment of the OSK-HIV-TS as would studies conducted in different contexts and among different subsets of the Brazilian population.
